# INCREASING CHILDREN’S EDUCATION IMPROVES PARENTAL DEMENTIA RISK AND REDUCES GRADIENTS IN RISK

**DOI:** 10.1093/geroni/igae098.2876

**Published:** 2024-12-31

**Authors:** Liying Luo, John Warren, Jiahui Xu, Kenneth Langa

**Affiliations:** The Pennsylvania State University, University Park, Pennsylvania, United States; University of Minnesota, Minneapolis, Minnesota, United States; The Pennsylvania State University, University Park, Pennsylvania, United States; University of Michigan, Ann Arbor, Michigan, United States

## Abstract

Recent research suggests that adult children’s education may influence their parents’ health at older ages. The empirical evidence about this upstream influence remains somewhat mixed, however. This is due in part to differing methodological approaches. To account for intergenerational selection and interdependent pathways, we use multilevel marginal structural models and well-defined casual estimands to analyze longitudinal data from the Health and Retirement study. We ask two important questions: (1) At the individual level, for whom does children’s education convey health benefits in older ages? (2) At the population level, what are the implications of increasing education levels among younger generations for (a) the health and well being of their parents’ generation and (b) educational disparities in health and well being? Our analyses suggests that at the individual level, increasing levels of children’s education decreases parents’ dementia risk. At the population level, educational disparities in dementia risk may be reduced as younger generations obtain more education.

